# Analysis of Prescriptions for Dual Antiplatelet Therapy After Acute Ischemic Stroke

**DOI:** 10.1001/jamanetworkopen.2022.24157

**Published:** 2022-07-28

**Authors:** Ying Xian, Haolin Xu, Roland Matsouaka, Daniel T. Laskowitz, Lesley Maisch, Deidre Hannah, Eric E. Smith, Gregg C. Fonarow, Deepak L. Bhatt, Lee H. Schwamm, Brian Mac Grory, Wuwei Feng, Emil Loldrup Fosbøl, Eric D. Peterson, Mark Johnson

**Affiliations:** 1 Department of Neurology, University of Texas Southwestern Medical Center, Dallas; 2 Duke Clinical Research Institute, Duke University, Durham, North Carolina; 3 Department of Neurology, Duke University School of Medicine, Durham, North Carolina; 4 Patient Co-Investigator; 5 Department of Clinical Neurosciences and Hotchkiss Brain Institute, University of Calgary, Calgary, Alberta, Canada; 6 Division of Cardiology, University of California at Los Angeles, Los Angeles; 7 Division of Cardiovascular Medicine, Brigham and Women’s Hospital, Harvard Medical School, Boston, Massachusetts; 8 Department of Neurology, Massachusetts General Hospital and Harvard Medical School, Boston; 9 Department of Cardiology, University Hospital of Copenhagen, Rigshospitalet, Copenhagen, Denmark; 10 Division of Cardiology, University of Texas Southwestern Medical Center, Dallas

## Abstract

**Importance:**

After the publication of the CHANCE (Clopidogrel in High Risk Patients With Acute Nondisabling Cerebrovascular Events) and POINT (Platelet-Oriented Inhibition in New Transient Ischemic Attack and Minor Ischemic Stroke) clinical trials, the American Heart Association/American Stroke Association (AHA/ASA) issued a new class 1, level of evidence A, recommendation for dual antiplatelet therapy (DAPT; aspirin plus clopidogrel) for secondary prevention in patients with minor ischemic stroke (National Institutes of Health Stroke Scale [NIHSS] score ≤3). The extent to which variations in DAPT prescribing patterns remain and the extent to which practice patterns in the US are consistent with evidence-based guidelines are unknown.

**Objective:**

To evaluate the discharge DAPT prescribing patterns after publication of the new AHA/ASA guidelines and assess the extent of hospital-level variation in the use of DAPT for secondary prevention in patients with minor stroke (NIHSS score ≤3), as indicated by guidelines, and in patients with nonminor stroke (NIHSS score >3), for whom the risks and benefits of DAPT have not been fully established.

**Design, Setting, and Participants:**

This multicenter retrospective cohort study involved 132 817 patients from 1890 hospitals participating in the AHA/ASA Get With The Guidelines–Stroke program. Patients who were hospitalized for acute ischemic stroke and prescribed antiplatelet therapy at discharge between October 1, 2019, and June 30, 2020, were included.

**Exposures:**

Minor ischemic stroke (NIHSS score ≤3) vs nonminor ischemic stroke (NIHSS score >3).

**Main Outcomes and Measures:**

The primary outcome was DAPT prescription at discharge. The extent to which variations in DAPT use were explained at the hospital level was assessed by calculating the median odds ratio (OR), which was derived using multivariable logistic regression analysis and compared the likelihood that 2 patients with identical clinical features admitted to 2 randomly selected hospitals (1 with higher propensity and 1 with lower propensity for DAPT use) would receive DAPT at discharge. Associations between hospital-level DAPT use among patients with minor vs nonminor stroke were evaluated using Pearson ρ correlation coefficients.

**Results:**

Among 132 817 patients (median [IQR] age, 68 [59-78] years; 68 768 men [51.8%]), 4282 (3.2%) were Asian, 11 254 (8.5%) were Hispanic, 27 221 (20.5%) were non-Hispanic Black, 84 468 (63.6%) were non-Hispanic White, and 5592 (4.2%) were of other races and/or ethnicities (including American Indian or Alaska Native, Native Hawaiian or Pacific Islander, and unable to determine). Overall, 86 551 patients (65.2%) presented with minor ischemic stroke, and 46 266 patients (34.8%) presented with nonminor ischemic stroke. After the 2019 AHA/ASA guideline updates, 40 661 patients (47.0%) with minor stroke (NIHSS median [IQR] score, 1 [0-2]) and 19 703 patients (42.6%) with nonminor stroke (NIHSS median [IQR] score, 6 [5-9]) received DAPT at discharge. Despite guideline recommendations, 45 890 patients (53.0%) with minor stroke did not receive DAPT. After accounting for patient characteristics, substantial hospital-level variations were found in the use of DAPT in those with minor stroke (median [IQR] hospital-level DAPT prescription rate, 44.8% [33.7%-57.7%]; range, 0%-91.7%; median OR, 2.03 [95% CI, 1.97-2.09]) when comparing 2 patients with identical risk factors discharged from 2 randomly selected hospitals, 1 with higher propensity and 1 with lower propensity for DAPT use. The use of DAPT in patients with nonminor stroke also varied significantly (median [IQR] hospital-level DAPT prescription rate, 41.4% [30.0%-53.8%]; range, 0%-100%; median OR, 1.90 [95% CI, 1.83-1.97]). Overall, hospitals that were more likely to prescribe DAPT for minor strokes were also more likely to prescribe DAPT for nonminor strokes (Pearson ρ = 0.72; *P* < .001).

**Conclusions and Relevance:**

This cohort study found that despite updated AHA/ASA guidelines, more than 50% of patients with minor acute ischemic stroke did not receive DAPT at discharge. In contrast, more than 40% of patients with nonminor stroke received DAPT despite lack of evidence in this setting. These findings suggest that enhancing adherence to evidence-based DAPT practice guidelines may be a target for quality improvement in the treatment of patients with ischemic stroke.

## Key Points

**Question:**

Have prescribing patterns in dual antiplatelet therapy (DAPT) for secondary prevention among patients with acute ischemic stroke changed after clinical trial findings and American Heart Association/American Stroke Association practice guideline updates?

**Findings:**

In this cohort study of 132 817 patients with acute ischemic stroke, 47.0% of patients with minor stroke received DAPT at discharge, as indicated by guidelines; 42.6% patients with nonminor stroke, for whom the risks and benefits of DAPT have not been fully established, received DAPT at discharge, with substantial hospital variation across current US practice.

**Meaning:**

This study’s findings suggest that enhancing adherence to evidence-based DAPT practice guidelines may be a target for quality improvement in the treatment of patients with ischemic stroke.

## Teaser

This cohort study used data from the American Heart Association/American Stroke Association Get With The Guidelines–Stroke registry to assess prescribing patterns for dual antiplatelet therapy at discharge among patients with minor and nonminor acute ischemic stroke.

## Introduction

Long-term dual antiplatelet therapy (DAPT) with aspirin and clopidogrel is not recommended for routine secondary prevention after ischemic stroke. However, from 2013 to 2018, evidence has increasingly supported the use of short-term (21-90 days) DAPT in patients with minor ischemic stroke (National Institutes of Health Stroke Scale [NIHSS] score ≤3) or high-risk transient ischemic attack (TIA).^1,2^ Based on findings from the CHANCE (Clopidogrel in High Risk Patients With Acute Nondisabling Cerebrovascular Events)^1^ and POINT (Platelet-Oriented Inhibition in New TIA and Minor Ischemic Stroke)^2^ clinical trials, the American Heart Association/American Stroke Association (AHA/ASA) issued a new class 1 (strong recommendation), level of evidence A (high-quality evidence from >1 randomized clinical trial [RCT], meta-analyses of high-quality RCTs, or ≥1 RCT corroborated by high-quality registry studies), recommendation for short-term use of DAPT for 21 days in patients presenting with minor noncardioembolic ischemic stroke (NIHSS score ≤3) or high-risk TIA in 2019,^3^ which was updated to use of DAPT for 21 to 90 days in 2021.^4^

Although DAPT is recommended only for specific patients with minor ischemic stroke and high-risk TIA, a recent study^5^ found substantial underuse of evidence-based DAPT in patients with minor ischemic stroke and potential overuse of DAPT in patients with nonminor ischemic stroke in US practice. Some variation in DAPT use may be expected based on an individual’s risk profile or preferences; however, the extent to which meaningful variations remain after accounting for patient characteristics is unknown. This information is important to develop a true understanding of the extent to which practice patterns are inconsistent with evidence-based guidelines. Therefore, we analyzed data from the AHA/ASA Get With The Guidelines–Stroke (GWTG-Stroke) registry to (1) quantify the proportion of patients with minor and nonminor acute ischemic stroke who received discharge DAPT prescriptions after the release of new AHA/ASA guideline recommendations between October 1, 2019, and June 30, 2020; (2) identify patient and hospital factors associated with DAPT use; (3) quantify the hospital-level variation in DAPT use after accounting for patient characteristics; and (4) evaluate the correlation between hospital-level DAPT use in patients with minor stroke vs nonminor stroke.

## Methods

This cohort study was approved by the institutional review board of Duke University. Each participating GWTG-Stroke hospital received either human research approval to enroll patients without individual informed consent under the Common Rule^6^ or a waiver of authorization and exemption from subsequent review by their institutional review boards. This study followed the Strengthening the Reporting of Observational Studies in Epidemiology (STROBE) reporting guideline for cohort studies.

### GWTG-Stroke Registry

The GWTG-Stroke registry is an ongoing voluntary national stroke registry sponsored by the AHA/ASA. Details of GWTG-Stroke registry data collection and variable definitions have been described previously.^7^ In brief, trained hospital personnel use an online patient management tool to collect data on consecutive patients with ischemic stroke admitted to each participating hospital. The eligibility of each stroke admission is confirmed through medical records review. Standardized data collection includes patient demographic characteristics, medical history, medications received before admission, diagnostic testing, brain imaging, in-hospital treatment, in-hospital outcomes, and medications prescribed at discharge. The validity and reliability of data collection have been reported previously.^8^ IQVIA Inc (Parsippany, New Jersey) serves as the GWTG-Stroke data collection and coordination center. The Duke Clinical Research Institute serves as the GWTG-Stroke data analysis center and has an agreement to analyze the aggregate deidentified data for research purposes.

### Study Population

**Figure 1.**
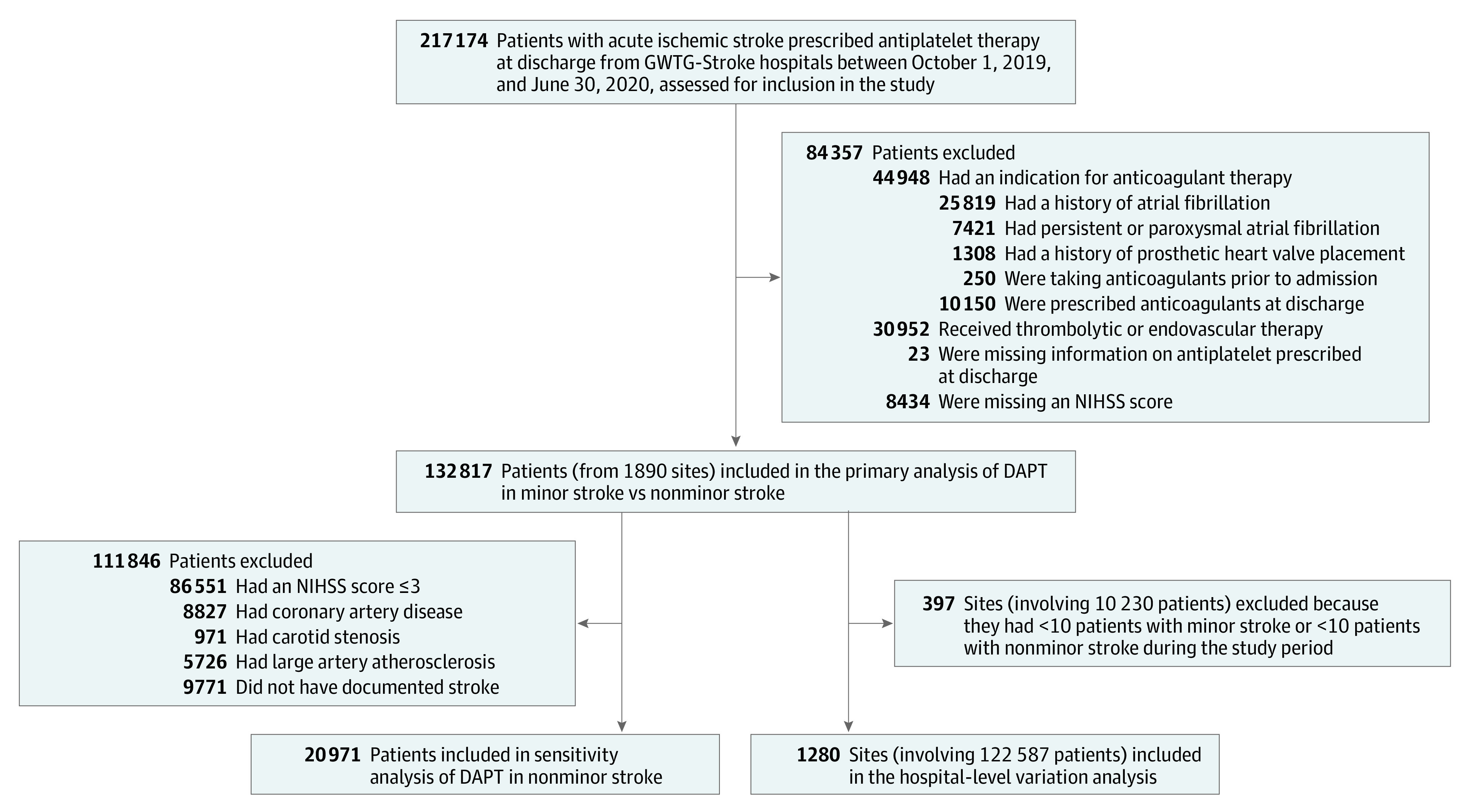
Study Population DAPT indicates dual antiplatelet therapy; GWTG-Stroke, Get With The Guidelines–Stroke program; and NIHSS, National Institutes of Health Stroke Scale.

This cohort study was a retrospective analysis of patients admitted for acute ischemic stroke and prescribed antiplatelet therapy at discharge from participating GWTG-Stroke hospitals in the US after the release of the 2019 AHA/ASA guidelines in October 2019^3^ and before the publication of the THALES (Ticagrelor and Aspirin for Prevention of Stroke and Death) clinical trial^9^ in June 2020. Patients hospitalized for elective carotid intervention only, such as carotid endarterectomy or stent, and patients presenting with TIA were not included. Additional details about inclusion and exclusion criteria are shown in Figure 1. In brief, we excluded patients with indications for anticoagulant therapy, such as those with atrial fibrillation or a prosthetic cardiac valve, those who received anticoagulant therapy before their stroke, and those who received a prescription for an anticoagulant at discharge. We further excluded patients who received thrombolytic or endovascular therapy because these therapies were part of the CHANCE^1^ and POINT^2^ exclusion criteria and patients who were missing information on NIHSS score and the type of antiplatelet therapy prescribed at discharge.

Antiplatelet agents were categorized into 5 groups: (1) aspirin alone; (2) clopidogrel alone; (3) DAPT with aspirin and clopidogrel; (4) aspirin and dipyridamole; and (5) other antiplatelet agents, such as ticlopidine, prasugrel, or ticagrelor, either alone or in combination with aspirin. Although DAPT technically refers to a combination of any 2 antiplatelet agents, for the purposes of the current study, DAPT denoted the combination of aspirin and clopidogrel. Ticlopidine is rarely used in current clinical practice owing to a higher risk of severe adverse effects, and prasugrel is contraindicated in patients with stroke. Although the 2021 AHA/ASA secondary prevention guidelines^4^ issued a new class 2b (weak recommendation), level of evidence B-R (moderate-quality evidence from ≥1 RCT or meta-analyses of moderate-quality RCTs), recommendation for ticagrelor plus aspirin for patients with minor to moderate stroke (NIHSS score ≤5) after the publication of the THALES clinical trial,^9^ ticagrelor alone and ticagrelor plus aspirin were not commonly used during our study period. Therefore, these antiplatelet agents were grouped together in the *other antiplatelet or combination therapy* category.

### Statistical Analysis

We calculated the proportion of patients with acute ischemic stroke who were prescribed DAPT at discharge according to NIHSS score (≤3 for minor stroke and >3 for nonminor stroke). Because DAPT may be used in certain clinical circumstances, such as severe symptomatic intracranial stenosis (class 2a [moderate recommendation], level of evidence B-NR [moderate-quality evidence from ≥1 nonrandomized, observational, or registry study or meta-analyses of such studies]), acute coronary syndrome, severe carotid stenosis, or large artery atherosclerosis, we also performed a sensitivity analysis (Figure 1) involving patients presenting with nonminor stroke (NIHSS score >3) by excluding individuals with a history of coronary artery disease, carotid stenosis, or large artery atherosclerosis stroke subtype and individuals without documented stroke etiology according to the TOAST (Trial of ORG 10172 in Acute Stroke Treatment) classification method.^10^

To identify factors associated with DAPT use, baseline patient and hospital characteristics were compared between patients who were prescribed vs not prescribed DAPT at discharge. To obtain a more reliable estimate of hospital-level variation of DAPT use in patients with minor and nonminor stroke, a variation analysis was performed that included only hospitals with at least 10 patients hospitalized for minor stroke and at least 10 patients hospitalized for nonminor stroke during the entire study period (Figure 1). Hospital-level DAPT use was reported (minimum use, 25th percentile, median, 75th percentile, and maximum use) by minor vs nonminor ischemic stroke. We then examined the extent to which variations in DAPT use were explained at the hospital level by calculating the median odds ratio (OR), derived using multivariable logistic regression analysis, with only patient-level factors included in the model.^11^ Patient-level variables included in the model were age (continuous), sex (male or female), race and ethnicity (Asian, Hispanic, non-Hispanic Black, non-Hispanic White, and other race and/or ethnicity [including American Indian or Alaska Native, Native Hawaiian or Pacific Islander, and unable to determine]), insurance status (Medicaid, Medicare, private, or self-pay), medical history (carotid stenosis, chronic kidney insufficiency, coronary artery disease or previous myocardial infarction, diabetes, dyslipidemia, heart failure, hypertension, peripheral vascular diseases, previous stroke, previous TIA, and smoking), medications received before admission (aspirin monotherapy, clopidogrel monotherapy, DAPT, aspirin and dipyridamole combination therapy, other antiplatelet or combination therapy, or unknown), and NIHSS score (range, 0-42, with higher scores indicating greater stroke severity) at admission.

Median OR was used to measure variation between the hospital-level DAPT prescription rate that could not be explained by patient factors alone. Median OR was calculated by comparing the likelihood that 2 patients with identical clinical features admitted to 2 randomly selected hospitals (1 with higher propensity and 1 with lower propensity for DAPT use) would be discharged with a prescription for DAPT. A median OR of 1.0 indicated no variation in DAPT use between hospitals, whereas a median OR greater than 1.0 suggested greater variation in DAPT use between hospitals after accounting for patient-level differences. The associations between hospital-level DAPT use in patients with minor vs nonminor stroke were reported graphically and evaluated using Pearson ρ correlation coefficients.

All statistical analyses were performed using SAS software, version 9.4 (SAS Institute Inc). Two-sided *P* < .05 was considered statistically significant.

## Results

A total of 132 817 patients with acute ischemic stroke (median [IQR] age, 68 [59-78] years; 68 768 men [51.8%] and 64 049 women [48.2%]) who were prescribed antiplatelet therapy at discharge from 1890 participating GWTG-Stroke hospitals between October 1, 2019, and June 30, 2020, were included in the primary analysis. Overall, 4282 patients (3.2%) were Asian, 11 254 (8.5%) were Hispanic, 27 221 (20.5%) were non-Hispanic Black, 84 468 (63.6%) were non-Hispanic White, and 5592 (4.2%) were of other races and/or ethnicities (including American Indian or Alaska Native, Native Hawaiian or Pacific Islander, and unable to determine).

The distribution of antiplatelet medication prescribed at discharge for secondary stroke prevention in patients with minor and nonminor ischemic stroke is shown in Table 1. In total, 86 551 patients (65.2%) presented with minor ischemic stroke (NIHSS score ≤3), and 46 266 patients (34.8%) presented with nonminor ischemic stroke (NIHSS score >3). Among patients with minor stroke (NIHSS median [IQR] score, 1 [0-2]), the most common antiplatelet regimen was DAPT (40 661 patients [47.0%]), followed by aspirin monotherapy (39 214 patients [45.3%]) and clopidogrel monotherapy (6176 patients [7.1%]). Despite guideline recommendations, 45 890 patients (53.0%) with minor stroke did not receive DAPT at discharge. Although the risks and benefits of DAPT use in patients with nonminor stroke have not been fully established, 19 703 patients (42.6%) presenting with nonminor stroke (NIHSS median [IQR] score, 6 [5-9]) received DAPT at discharge. In the sensitivity analysis excluding individuals with coronary artery disease, carotid stenosis, large artery atherosclerosis, and/or no documented stroke etiology, 7800 of 20 971 patients (37.2%) with nonminor stroke received DAPT at discharge.

**Table 1.** Antiplatelet Prescription Patterns After the American Heart Association/American Stroke Association 2019 Guideline Updates

Antiplatelet therapy at discharge	Patients, No. (%)	
	Minor stroke (NIHSS score ≤3)	Nonminor stroke (NIHSS score >3)
Total patients, No.	86 551	46 266
Aspirin monotherapy	39 214 (45.3)	22 791 (49.3)
Clopidogrel monotherapy	6176 (7.1)	3478 (7.5)
DAPT (aspirin and clopidogrel)	40 661 (47.0)	19 703 (42.6)
Aspirin and dipyridamole combination therapy	343 (0.4)	197 (0.4)
Other antiplatelet or combination therapy	157 (0.2)	97 (0.2)

Abbreviations: DAPT, dual antiplatelet therapy; NIHSS, National Institutes of Health Stroke Scale.

Baseline characteristics of patients who were prescribed vs not prescribed DAPT at discharge, stratified by NIHSS score, are shown in Table 2. Among those with minor stroke, 40 661 patients (47.0%) were prescribed DAPT at discharge, and 45 890 (53.0%) were not. Among those with nonminor stroke, 19 703 patients (42.6%) were prescribed DAPT at discharge, and 26 563 (57.4%) were not. In general, when comparing DAPT vs no DAPT receipt among those with NIHSS scores ≤3 vs >3, male patients, non-Hispanic White patients, patients with cardiovascular risk factors, and patients receiving DAPT before their stroke were more likely to receive DAPT, regardless of NIHSS score.

**Table 2.** Baseline Characteristics, Stratified by NIHSS Score and DAPT Prescription at Discharge, After the American Heart Association/American Stroke Association 2019 Guideline Updates

Characteristic	Patients, No./total No. (%)			
	Minor stroke (NIHSS score ≤3)		Nonminor stroke (NIHSS score >3)	
	Prescribed DAPT at discharge	Not prescribed DAPT at discharge	Prescribed DAPT at discharge	Not prescribed DAPT at discharge
**Patient characteristics**				
Total patients, No.	40 661	45 890	19 703	26 563
Age, median (IQR), y	68 (59-77)	68 (58-77)	69 (60-78)	69 (59-79)
Sex				
Female	18 046/40 661 (44.4)	22 633/45 890 (49.3)	9439/19 703 (47.9)	13 931/26 563 (52.4)
Male	22 615/40 661 (55.6)	23 257/45 890 (50.7)	10 264/19 703 (52.1)	12 632/26 563 (47.6)
Race and ethnicity				
Asian	1208/40 661 (3.0)	1493/45 890 (3.3)	634/19 703 (3.2)	947/26 563 (3.6)
Hispanic	3147/40 661 (7.7)	4046/45 890 (8.8)	1615/19 703 (8.2)	2446/26 563 (9.2)
Non-Hispanic Black	6961/40 661 (17.1)	8727/45 890 (19.0)	4688/19 703 (23.8)	6845/26 563 (25.8)
Non-Hispanic White	27 775/40 661 (68.3)	29 742/45 890 (64.8)	11 876/19 703 (60.3)	15 075/26 563 (56.8)
Other^a^	1570/40 661 (3.9)	1882/45 890 (4.1)	890/19 703 (4.5)	1250/26 563 (4.7)
Insurance status				
Medicaid	4013/34 616 (11.6)	5018/38 954 (12.9)	3043/16 809 (18.1)	4381/22 748 (19.3)
Medicare	14 091/34 616 (40.7)	15 271/38 954 (39.2)	7286/16 809 (43.3)	9603/22 748 (42.2)
Private	14 656/34 616 (42.3)	16 197/38 954 (41.6)	5620/16 809 (33.4)	7401/22 748 (32.5)
Self-pay	1856/34 616 (5.4)	2468/38 954 (6.3)	860/16 809 (5.1)	1363/22 748 (6.0)
NIHSS score, median (IQR)	1 (0-2)	1 (0-2)	6 (5-9)	7 (5-11)
Medical history				
CAD or previous MI	9064/40 661 (22.3)	6444/45 890 (14.0)	4666/19 703 (23.7)	4161/26 563 (15.7)
Carotid stenosis	2004/40 661 (4.9)	1080/45 890 (2.4)	1039/19 703 (5.3)	679/26 563 (2.6)
Chronic kidney insufficiency	3766/40 661 (9.3)	4080/45 890 (8.9)	2212/19 703 (11.2)	2715/26 563 (10.2)
Diabetes	16 633/40 661 (40.9)	15 704/45 890 (34.2)	9147/19 703 (46.4)	10 063/26 563 (37.9)
Dyslipidemia	22 377/40 661 (55.0)	20 844/45 890 (45.4)	10 720/19 703 (54.4)	11 683/26 563 (44.0)
Heart failure	2189/40 661 (5.4)	2107/45 890 (4.6)	1495/19 703 (7.6)	1845/26 563 (6.9)
Hypertension	32 053/40 661 (78.8)	33 917/45 890 (73.9)	16 142/19 703 (81.9)	20 195/26 563 (76.0)
Peripheral vascular disease	1712/40 661 (4.2)	1123/45 890 (2.4)	952/19 703 (4.8)	740/26 563 (2.8)
Previous stroke	9895/40 661 (24.3)	7958/45 890 (17.3)	7280/19 703 (36.9)	7174/26 563 (27.0)
Previous TIA	3850/40 661 (9.5)	2885/45 890 (6.3)	1655/19 703 (8.4)	1687/26 563 (6.4)
Smoking	8963/40 661 (22.0)	9675/45 890 (21.1)	4949/19 703 (25.1)	6133/26 563 (23.1)
Antiplatelet therapy before admission				
Aspirin monotherapy	13 854/40 661 (34.1)	12 896/45 890 (28.1)	6272/19 703 (31.8)	7896/26 563 (29.7)
Clopidogrel monotherapy	2038/40 661 (5.0)	1331/45 890 (2.9)	1258/19 703 (6.4)	990/26 563 (3.7)
DAPT (aspirin and clopidogrel)	5598/40 661 (13.8)	965/45 890 (2.1)	3505/19 703 (17.8)	794/26 563 (3.0)
Aspirin and dipyridamole combination therapy	81/40 661 (0.2)	128/45 890 (0.3)	43/19 703 (0.2)	86/26 563 (0.3)
Other antiplatelet or combination therapy	173/40 661 (0.4)	494/45 890 (1.1)	80/19 703 (0.4)	295/26 563 (1.1)
Unknown	1/40 661 (<0.1)	12/45 890 (<0.1)	0	4 (<0.1)
**Hospital characteristics**				
Beds, median (IQR), No.	350 (225-538)	334 (213-512)	369 (235-562)	369 (235-562)
Academic center	30 054/40 661 (73.9)	32 580/45 890 (71.0)	14 863/19 703 (75.4)	19 914/26 563 (75.0)
Stroke center certification				
Primary stroke center	9057/40 661 (22.3)	9234/45 890 (20.1)	4965/19 703 (25.2)	6904/26 563 (26.0)
Comprehensive stroke center	22 447/40 661 (55.2)	26 702/45 890 (58.2)	10 328/19 703 (52.4)	14 192/26 563 (53.4)
Annual ischemic stroke volume, median (IQR), No	257 (172-393)	245 (167-366)	268 (183-410)	263 (178-398)
Region				
Midwest	8845/40 661 (21.8)	9332/45 890 (20.3)	4027/19 703 (20.4)	5259/26 563 (19.8)
Northeast	8410/40 661 (20.7)	8755/45 890 (19.1)	3728/19 703 (18.9)	4689/26 563 (17.7)
South	16 641/40 661 (40.9)	19 267/45 890 (42.0)	8624/19 703 (43.8)	11 468/26 563 (43.2)
West	6765/40 661 (16.6)	8536/45 890 (18.6)	3324/19 703 (16.9)	5147/26 563 (19.4)
Rural	1989/40 661 (4.9)	2275/45 890 (5.0)	1008/19 703 (5.1)	1265/26 563 (4.8)

Abbreviations: CAD, coronary artery disease; DAPT, dual antiplatelet therapy; MI, myocardial infarction; NIHSS, National Institutes of Health Stroke Scale; TIA, transient ischemic attack.

^a^ Other race and ethnicities include American Indian or Alaska Native, Native Hawaiian or Pacific Islander, or unable to determine.

**Figure 2.**
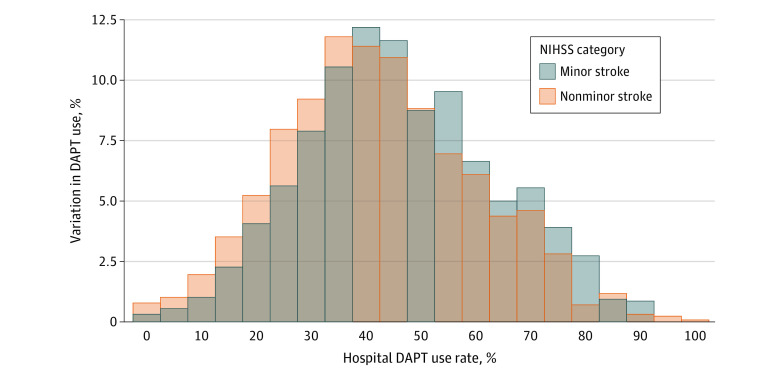
Hospital-Level Variations in DAPT Use Among Patients With Minor Stroke and Nonminor Stroke DAPT indicates dual antiplatelet therapy; and NIHSS, National Institutes of Health Stroke Scale.

**Figure 3.**
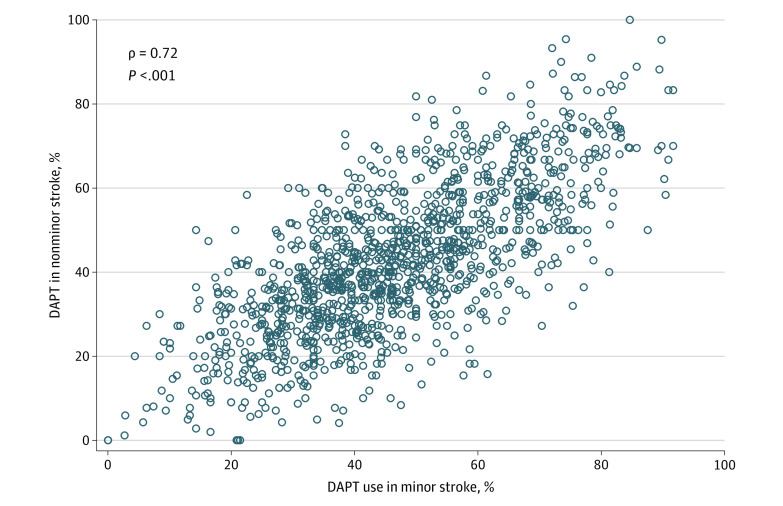
Correlation Between Hospital-Level DAPT Use Among Patients With Minor Stroke vs Nonminor Stroke DAPT indicates dual antiplatelet therapy.

Substantial hospital-level variations were found in the proportion of DAPT use in patients with minor vs nonminor stroke. At least 57.7% of patients with minor ischemic stroke received DAPT if admitted to hospitals in the highest quartile (75th percentile to maximum) of DAPT use compared with 33.7% or fewer if admitted to hospitals in the lowest quartile (minimum to 25th percentile) of DAPT use (median hospital-level DAPT prescription rate, 44.8%; range, 0%-91.7%) (Figure 2). In other words, among hospitals in the highest quartile of DAPT use, up to 42.3% of patients with minor stroke did not receive DAPT for secondary prevention; among hospitals in the lowest quartile of DAPT use, more than 66.3% of patients with minor stroke were discharged without a prescription for DAPT. In addition, at least 53.8% of patients with nonminor stroke received DAPT at discharge if admitted to hospitals in the highest quartile of DAPT use compared with 30.0% or fewer if admitted to hospitals in the lowest quartile of DAPT use (median hospital-level DAPT prescription rate, 41.4%; range, 0%-100%). After accounting for observed patient characteristics, the median OR was 2.03 (95% CI, 1.97-2.09) for those with minor stroke. That is, if the same patient with minor ischemic stroke was randomly admitted to a hospital with a higher vs lower propensity for DAPT use, the odds of receiving DAPT were slightly more than 2.0-fold higher. The median OR for those with nonminor ischemic stroke was 1.90 (95% CI, 1.83-1.97). That is, if the same patient with nonminor ischemic stroke was randomly admitted to a hospital with a higher vs lower propensity for DAPT use, the odds of receiving DAPT (potentially inappropriately) were 1.9-fold higher. A significant positive correlation was found between hospital-level DAPT use among patients with minor stroke vs nonminor stroke (Pearson ρ = 0.72; *P* < .001) (Figure 3). In other words, hospitals that were more (or less) likely to prescribe DAPT to patients with minor ischemic stroke were also more (or less) likely to prescribe DAPT to patients with nonminor ischemic stroke.

## Discussion

In this large cohort study of patients with acute ischemic stroke in the US, 53.0% of patients with minor ischemic stroke did not receive DAPT for secondary stroke prevention, even after publication of the new AHA/ASA class 1, level of evidence A, recommendation in October 2019.^3^ In contrast, 42.6% of patients with nonminor ischemic stroke who did not meet the CHANCE^1^ or POINT^2^ eligibility criteria received DAPT, even though the risk-benefit ratio of DAPT in such settings has not been fully established. In addition, there were substantial hospital-level variations in the use of DAPT among patients with minor vs nonminor stroke, which could not be explained by differences in patient-level factors alone. Although the NIHSS score is a major factor associated with DAPT eligibility, hospitals that were more (or less) likely to prescribe DAPT for secondary prevention in patients with minor stroke were also more (or less) likely to prescribe DAPT for secondary prevention in patients with nonminor stroke, suggesting indiscriminate prescription of DAPT or single antiplatelet agents in certain hospitals. Taken together, these findings suggest an important opportunity to improve the use of evidence-based antiplatelet therapy for secondary stroke prevention in patients with acute ischemic stroke.

Antiplatelet therapy plays an important role in secondary prevention among patients with acute ischemic stroke. The most commonly used antiplatelet agents are aspirin, clopidogrel, and DAPT with aspirin and clopidogrel. In 2004, the MATCH (Management of Atherothrombosis With Clopidogrel in High-Risk Patients With Recent Transient Ischemic Attacks or Ischemic Stroke) clinical trial^12^ examined the use of DAPT vs clopidogrel monotherapy in patients with recent stroke and found no significant benefit to treatment with DAPT and an increased risk of major bleeding complications. After publication of the MATCH clinical trial,^12^ a rapid reduction in DAPT use was observed across the US.^13^ Unlike the MATCH study^12^ and other secondary prevention clinical trials,^14,15,16^ the CHANCE clinical trial^1^ assessed the use of DAPT for 21 days, and the POINT clinical trial^2^ assessed the use of DAPT for 90 days in patients with minor stroke (NIHSS score ≤3) or high-risk TIA; both studies demonstrated a significant benefit of DAPT, with no increase in bleeding in the CHANCE clinical trial^1^ but higher risk of major bleeding in the POINT clinical trial.^2^ In response to these findings, the AHA/ASA issued a new class 1, level of evidence A, recommendation based on the CHANCE^1^ and POINT^2^ eligibility criteria for DAPT use in patients with minor noncardioembolic ischemic stroke (NIHSS score ≤3) in 2019.^3,17,18^

A previous study^5^ reported a rapid and sustained change in DAPT use that immediately coincided with the publication of pivotal clinical trials and new AHA/ASA guideline recommendations. Although these findings suggested that changes in physician prescribing behavior occurred in response to the new knowledge,^5^ the translation of evidence to clinical practice has been incomplete; as many as 53.0% of patients with minor stroke in our study did not receive DAPT at discharge. On the other hand, we found increasing adoption of DAPT in patients presenting with nonminor ischemic stroke who did not meet the CHANCE^1^ or POINT^2^ eligibility criteria. Although some physicians may extend DAPT use to patients with NIHSS scores of 5 or less, intracranial large artery atherosclerosis, or severe stenosis, the risks and benefits of DAPT have not been well established.^19^ Given that less-intensive antiplatelet therapy may increase the risk of recurrent ischemic events, and combination therapy may increase the risk of bleeding complications,^4,20^ the consequences of potential underuse of DAPT in patients with minor stroke and the use of DAPT in patients with nonminor stroke need to be assessed in future research.

Some clinicians may avoid prescribing DAPT to patients with minor stroke in the presence of an allergy to aspirin or clopidogrel, a known bleeding diathesis or history of bleeding, a large territory infarction despite a low NIHSS score, a high risk of bleeding, a risk of fall, or *CYP2C19* polymorphism. The use of DAPT in patients with nonminor stroke may be pursued in the setting of carotid artery stenting, recent percutaneous coronary intervention, aortic arch atherosclerosis, intracranial large artery atherosclerosis, severe symptomatic intracranial stenosis, or even patient preferences.^21,22,23,24,25,26,27,28,29^ Despite a lack of evidence,^19^ these extenuating circumstances may represent the normal variations inherent in medical practice rather than nonadherence to guidelines. Furthermore, certain practitioners may choose to intensify antithrombotic therapy from aspirin to DAPT for patients who experience breakthrough strokes while receiving aspirin therapy.^30^ Although the safety and efficacy of changing from a single antiplatelet agent to DAPT have not been established,^4^ the present study controlled for previous stroke and previous medication receipt before hospital admission. Notably, our study findings were essentially unchanged in the sensitivity analysis excluding patients with coronary artery disease, carotid stenosis, or large artery atherosclerosis. Even if DAPT was prescribed specifically for patients with large artery atherosclerosis, DAPT is only recommended for very specific patients who have had a recent stroke associated with severe symptomatic intracranial stenosis (ie, 70%-99% stenosis; class 2a, level of evidence B-NR).^4^

It should be noted that support for the use of DAPT among patients with severe intracranial atherosclerotic stenosis is based on data from the CLAIR (Clopidogrel Plus Aspirin for Infarction Reduction) clinical trial,^21^ a post hoc analysis of the CHANCE clinical trial,^31^ and the SAMMPRIS (Stenting and Aggressive Medical Management for Preventing Recurrent Stroke in Intracranial Stenosis) clinical trial.^27^ Caution is warranted when interpreting the results of the SAMMPRIS clinical trial^27^ because the study compared DAPT (accompanied by a suite of risk factor–modifying interventions) with percutaneous transluminal angioplasty and stenting. A benefit to DAPT in that clinical trial^27^ was only inferred based on a comparison of the medical arm with historical controls. The CLAIR clinical trial^21^ specifically excluded individuals with more severe stroke (NIHSS score >8), and the CHANCE clinical trial^31^ excluded patients with NIHSS scores greater than 3. Despite a pattern of lower event rates with the use of DAPT, neither the CLAIR clinical trial^21^ nor an underpowered subgroup analysis of the CHANCE clinical trial^31^ found a statistically significant difference between the use of DAPT vs aspirin alone in terms of preventing subsequent stroke. Notably, the present study found that even if DAPT were prescribed specifically for patients with nonminor stroke who have NIHSS scores greater than 3 and NIHSS scores of 5 or less, three-quarters of DAPT use in patients with nonminor stroke occurred among those with NIHSS scores greater than 5 (median [IQR] score, 6 [5-9]). Therefore, these factors are unlikely to account for most of the DAPT use among patients with nonminor stroke observed in our data.

The rates of DAPT use in patients with minor vs nonminor stroke varied markedly at the hospital level, even after accounting for patient-level characteristics. Although some level of variation may be associated with unmeasured patient factors (allergy to aspirin or clopidogrel, known bleeding diathesis or history of bleeding, large territory infarction despite low NIHSS score, high bleeding risk, risk of fall, *CYP2C19* polymorphism, carotid artery stenting, recent percutaneous coronary intervention, aortic arch atherosclerosis, intracranial large artery atherosclerosis, severe symptomatic intracranial stenosis, or patient preferences), there is no reason to believe that the proportion of such exceptional cases would vary substantially across hospitals. Even in the hospitals in the highest quartile of DAPT use, up to 42.3% of patients with minor strokes did not receive DAPT for secondary prevention, whereas in hospitals in the lowest quartile of DAPT use, more than 66.3% of patients with minor strokes were discharged without a prescription for DAPT. Notably, hospitals that were more (or less) likely to prescribe DAPT for secondary prevention in those with minor stroke were also more (or less) likely to prescribe DAPT for secondary prevention in those with nonminor stroke. Although specific recommendations for the use of DAPT exist, it appears that they were not closely followed in clinical practice, and physicians may use a one-size-fits-all approach to prescribing antiplatelet therapy for secondary stroke prevention. This substantial gap suggests that DAPT prescribing for patients with minor ischemic stroke may be used as a guideline-based performance measure, representing a compelling quality improvement target for the treatment of ischemic stroke. Furthermore, the substantial hospital variation in rates of DAPT use in patients with nonminor stroke represents gaps in knowledge and highlights the need for future clinical trials to clarify the risks and benefits of DAPT for secondary prevention in patients presenting with nonminor ischemic stroke.^19^ With more than 690 000 ischemic strokes occurring in the US each year,^32^ developing evidence-based approaches to the use of antiplatelet therapy and improving adherence to evidence-based practice guidelines would yield substantial health benefits for this vulnerable population.^33^

### Limitations

This study has limitations. First, the study was a retrospective observational analysis. Despite containing a large number of clinical details, including medical history, previous stroke medications, and NIHSS score at presentation, the GWTG-Stroke registry does not document the reasons or specific clinical circumstances for prescribing vs not prescribing DAPT. Therefore, treatment selection and unmeasured confounding could have had consequences for the validity of study findings. Second, the current guidelines recommend that DAPT be ideally initiated within 12 to 24 hours but no later than 7 days after symptom onset and continued for 21 days. The GWTG-Stroke registry does not have information on timing of the initiation and duration of antiplatelet treatment. Although uncommon, it is possible that DAPT was initiated and discontinued for bleeding complications during hospitalization or that the length of hospital stay was longer than 21 days.

Third, our analysis focused on hospital variation in DAPT use. We were unable to analyze practitioner variation. It is likely that practitioner variation exists, even within the same hospital. Fourth, the THALES clinical trial,^9^ which was published in July 2020, extended the benefit of short-term DAPT with ticagrelor beyond patients with minor stroke to include a portion of patients who presented with more severe deficits (NIHSS score ≤5). The AHA/ASA subsequently issued a class 2b, level of evidence B-R, recommendation of ticagrelor plus aspirin for patients with minor to moderate stroke (NIHSS score ≤5).^4^ Our study was conducted before the publication of the THALES clinical trial. Neither ticagrelor nor ticagrelor plus aspirin were commonly used during our study period. Future research is needed to evaluate the impact of the THALES clinical trial^9^ findings for secondary stroke prevention. Fifth, our study analyzed antiplatelet prescription patterns in participating GWTG-Stroke hospitals. Despite being the largest stroke registry, covering more than three-quarters of the US population, these results might not be applicable for extrapolation to patients receiving treatment at nonparticipating GWTG-Stroke hospitals. That said, many GWTG-Stroke hospitals are large academic centers, rates of adherence to evidence-based DAPT could be lower, and hospital variation may be even larger in nonparticipating hospitals.

## Conclusions

In this large cohort study using data from a national registry, a substantial proportion of patients with acute ischemic stroke did not receive appropriate antiplatelet therapy, and there were wide variations in DAPT use across hospitals nationwide. Enhancing adherence to evidence-based DAPT practice guidelines may be a target for quality improvement in the treatment of patients with ischemic stroke.
